# Antiviral activity of a novel mixture of natural antimicrobials, in vitro, and in a chicken infection model in vivo

**DOI:** 10.1038/s41598-020-73916-1

**Published:** 2020-10-06

**Authors:** Igori Balta, Lavinia Stef, Ioan Pet, Patrick Ward, Todd Callaway, Steven C. Ricke, Ozan Gundogdu, Nicolae Corcionivoschi

**Affiliations:** 1 Bacteriology Branch, Veterinary Sciences Division, Agri-Food and Biosciences Institute, 18a Newforge Lane, Belfast, BT9 5PX Northern Ireland, UK; 2 Faculty of Animal Science and Biotechnologies, University of Agricultural Sciences and Veterinary Medicine, 400372 Cluj-Napoca, Romania; 3 Faculty of Bioengineering of Animal Resources, Banat University of Animal Sciences and Veterinary Medicine - King Michael I of Romania, Timisoara, Romania; 4 Auranta, Nova UCD, Belfield, Dublin 4, Ireland; 5 Department of Animal and Dairy Science, University of Georgia, Athens, GA USA; 6 Center for Food Safety, Department of Food Science, University of Arkansas, Fayetteville, AR USA; 7 Faculty of Infectious and Tropical Diseases, London School of Hygiene and Tropical Medicine, 13 Keppel Street, London, WC1E 7HT UK

**Keywords:** Viral host response, Chemokines

## Abstract

The aim of this study was to test in vitro the ability of a mixture of citrus extract, maltodextrin, sodium chloride, lactic acid and citric acid (AuraShield L) to inhibit the virulence of infectious bronchitis, Newcastle disease, avian influenza, porcine reproductive and respiratory syndrome (PRRS) and bovine coronavirus viruses. Secondly, in vivo*,* we have investigated its efficacy against infectious bronchitis using a broiler infection model. In vitro, these antimicrobials had expressed antiviral activity against all five viruses through all phases of the infection process of the host cells. In vivo, the antimicrobial mixture reduced the virus load in the tracheal and lung tissue and significantly reduced the clinical signs of infection and the mortality rate in the experimental group E2 receiving AuraShield L. All these effects were accompanied by a significant reduction in the levels of pro-inflammatory cytokines and an increase in IgA levels and short chain fatty acids (SCFAs) in both trachea and lungs. Our study demonstrated that mixtures of natural antimicrobials, such AuraShield L, can prevent in vitro viral infection of cell cultures. Secondly, in vivo, the efficiency of vaccination was improved by preventing secondary viral infections through a mechanism involving significant increases in SCFA production and increased IgA levels. As a consequence the clinical signs of secondary infections were significantly reduced resulting in recovered production performance and lower mortality rates in the experimental group E2.

## Introduction

Viral infections are increasingly frequent in humans and farm animals and because of their ability to suffer genetic modifications in a relatively short period of time there is a constant need to develop novel pharmaceuticals in order to increase the efficacy of treatment^1^. Some of the most common viral diseases in farmed animals are infectious bronchitis, Newcastle disease, avian influenza, porcine reproductive and respiratory syndrome (PRRS) and bovine coronavirus. Currently, vaccination for these diseases is only partially efficacious therefore additional preventions and treatments are needed.

Natural antimicrobials including plant extracts or organic acids are currently tested for their ability to inhibit viruses and prevent their pathogenic impact on the host. Plant phytochemicals, in particular flavonoids and polyphenols, contain an abundant pool of potent antiviral molecules (e.g., vitexin), a flavonoid isolated from pink coral tree *Erythrina speciose* leaves, demonstrated antiviral activity against Herpes Simplex Virus type 1 (HSV-1) and Hepatitis A virus-H10 (HAV H10)^2^. Improving the efficiency of antimicrobial compounds represents a crucial step in developing alternative strategies, but this depends on a better understanding of their mode of action. For example, gamma-coronavirus (IBV) that was pre-treated with elderberry (*Sambucus nigra*) fruit extracts resulted in damage to virus molecular structure causing an elimination of Vero cell cytotoxicity^3^. The mechanism of elderberry extract efficacy was attributed to altered virion envelopes and membrane vesicles^4^.

The identification of the active compounds with antiviral effects will lead to a better understanding on how natural antimicrobials inhibit viruses and diseases. Tangeretin is a polymethoxylated flavone found in citrus fruit peels that inhibits viral entry into cells by blocking viral fusion^5^, and citrus extracts are active against avian influenza virus (AIV), Newcastle disease virus (NDV), infectious bursal disease virus (IBDV) in different environments^6^. Moreover, vaccines containing citrus derived molecules were more efficient in stimulating the immune system with fewer side effects^7^. Maltodextrins are plant polysaccharides that are commonly used as food additives that when used as a vaccine nanoparticle increased activity against influenza virus^8^. Lactic acid also inhibited influenza A infection replication^8^. Citric acid is another potent natural antimicrobial with anti-viral activity that inhibits the foot and mouth disease virus (FMDV)^9^.

An additional anti-viral mechanism by which natural antimicrobials can prevent viral infections refers to their ability to inhibit cellular oxidative events. For example, manganese superoxide dismutase (MnSOD) has been proven crucial in the fight against viral infections due to its role as superoxide scavenger and as anti-inflammatory agent^10^. The pro-inflammatory response, including the release of cytokines to avoid apoptosis is partially dependent on the levels of cellular hydrogen peroxide production as it leads to the stimulation of neutrophils and macrophages leading to the elimination of either microbes or viruses^11^. Natural antimicrobials or plant extracts (phenolics, flavonoids, tannins) have the ability to negatively impact on oxidative stress helping the organism to protect itself from the detrimental effect of reactive oxygen species^12^.

Combinations of natural antimicrobials, similar to AuraShield L (e.g. Auranta 3001), were previously described as efficient in preventing bacterial infections in chicken and mouse animal models^13–16^. These results show that Auranta 3001 has a negative impact on the expression of virulence genes in *Campylobacter* spp.^13^, (*hcp* gene) and that improves the immune system response in challenged broilers. In infections caused by *Listeria* spp., these mixtures of antimicrobials were able to reduce the bacterial load in the liver and spleen of the infected mice and significantly reduced inflammation and reduced the mortality rate^15^. It is thought that these mixtures of natural biochemical antimicrobials can directly affect viruses through their viral capsids or modify the receptors on the host cells involved in adhesion being strongly suggested that a comprehensive understanding of their anti-viral mechanisms is strongly recommended^17^.

Understanding the mechanisms involved in the anti-viral and anti-infectious activity of biochemical compounds require extensive in vivo testing in order to prove their efficiency in reducing the clinical signs of disease without a negative impact on animal health. We hypothesized that given the fact that in combination natural antimicrobials can act antagonistically against each other^18^ a detailed scientific report is often required, allowing the end user to design informed interventions at farm level. In the present study we examined the in vitro activity of a mixture of citrus extract, maltodextrin, sodium chloride, lactic acid and citric acid (AuraShield L) against viruses causing infectious bronchitis (B1648), Newcastle disease (ATCC, 699), avian influenza (H9N2, ATCC, VR-1642), porcine reproductive and respiratory syndrome (ATCC, VR-2386) and bovine coronavirus (ATCC, VR-874). We have investigated the effectiveness of this antimicrobial mixture by supplementing it via drinking water, against the infectious bronchitis virus in challenged broilers (B1648).

## Results

### The in vitro antiviral effect of AuraShield L

**Figure 1:**
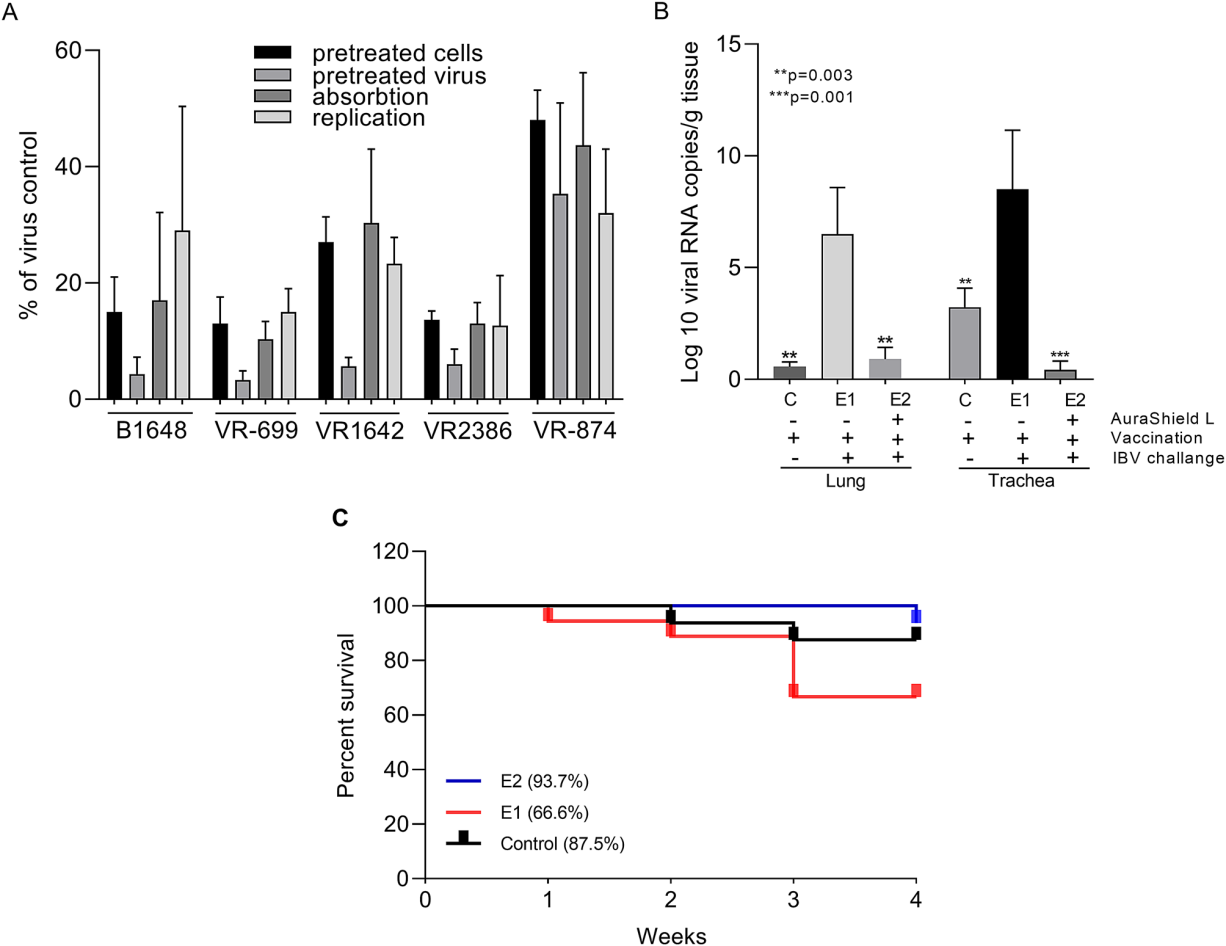
(**A**) the anti-viral effect of AuraShield L against B1648, ATCC, 699, VR-1642, VR-2386 and VR-874. AuraShield L was added at the non-cytotoxic concentration of 0.01%. Cells were pre-treated with AuraShield L prior to virus infection (pre-treatment cells), viruses were pre-treated prior to infection (pre-treated virus), AuraShield L was added during the adsorption period (adsorption) or after penetration of the viruses into cells (replication). Two-way ANOVA and Three-way ANOVA was used to analyse the significance of percentage decrease. (**B**) Viral RNA copies in the trachea and lungs. Viral RNA copies were measured by RT-qPCR. In (**C**) the average percentage of mortality in groups C, E1 and E2 is represented (birds per group at each time point). *Asterisks* indicate significant differences as indicated on the graph. *Error bars* represent the standard deviation of means from three replicate experiments.

First we have investigated, in vitro, as described in Materials and Methods the antiviral effect of AuraShield L against the five of the most common infectious viruses in livestock including Infectious Bronchitis (B1648), Newcastle disease (ATCC, 699), Avian Influenza (H9N2, ATCC, VR-1642), porcine reproductive and respiratory syndrome (ATCC, VR-2386) and bovine coronavirus (ATCC, VR-874). The 50% effective concentration (EC_50_) of AuraShield L was detected between 0.004 and 0.007 μl/ml, with the highest concentrations (0.007 μl) required against VR-2386 (Table 1). Next we have demonstrated the effect of the antimicrobial mixture on the viral replication by treating the cells and the viruses individually with AuraShield L prior to infection. Additionally, the antimicrobial mixture was tested by inclusion during adsorption or after the adsorption period. Infected cells in the absence of AuraShield L were used as a control. The percent reduction in viral replication was calculated relative to the amount of virus produced in controls, and a non-cytotoxic concentration was used in all assays. Pre-treatment with AuraShield L caused a decrease in the number of plaques in all experimental investigations and for all viruses used (Fig. 1A). Pre-treatment of cells with AuraShield L (0.01%) led to over 80% reduction in B1648, VR-699 and VR-2386 virus production compared to control. In the case of VR-1642 (Fig. 1A) the reduction was of approximately 70% and 50% for VR-874. The impact was even more dramatic when viruses were pre-treated with AuraShield L with over 90% reduction for all viruses except VR-874 (65%). When AuraShield L was added during the adsorption period, virus titres were reduced by 52% for VR-874. Similar results were observed during viral replication, when the antimicrobial mixture was added only to the overlay medium. Collectively, these results suggest that the anti-viral effect of AuraShield L is exerted through all phases of host cell infection.

**Table 1:** Cytotoxic and efficiency of AuraShield L.

Disease	Virus	CC_50_ (µl/ml)	EC_50_ (µl/ml)	SI
Avian infectious bronchitis	B1648	0.27	0.004	67.5
Newcastle disease	ATCC-699	0.33	0.004	82.5
Avian Influenza	VR-1642	0.40	0.006	66.6
Porcine reproductive and respiratory syndrome	VR-2386	0.54	0.007	77.1
Bovine coronavirus	VR-874	0.19	0.004	47.5

CC, cytotoxic concentration; EC, effective concentration; SI, selective index (CC_50_/EC_50_).

### AuraShield L reduces the viral load in the trachea and lungs of in vivo challenged broilers with the avian bronchitis virus (B1648)

We have next examined the anti-viral effect of AuraShield L in broilers in an in vivo infectious bronchitis model using the B1648 virus to cause infection. Viral RNA was measured in samples of tracheal and lung samples, using RT-qPCR (Fig. 1B). The viral RNA copies in the lung were at approximately 7.1 log_10_ copies/g tissue in group E1 with a significant decrease in the group E2 (*p* = 0.003) similar to the un-infected control group C. Inclusion of AuraShield L in the broiler’s drinking water reduced the viral load in the trachea from 8.7 log_10_ copies/g, in the untreated group E1, to 0.8 log_10_ copies/g in the tracheal tissue (*p* = 0.001) of the treated group E2. The virus levels were reduced below the levels recorded in the un-treated and un-challenged group C. The levels of significance were calculated considering group E1 as reference. These results indicate that the antimicrobial mixture reduced the virus load in the tracheal tissue having a negative impact the virus transition into the lung tissue.

### AuraShield L has a negative impact on the pro-inflammatory cytokines levels, stimulates immunity and reduces oxidative inflammation in challenged broilers

**Figure 2:**
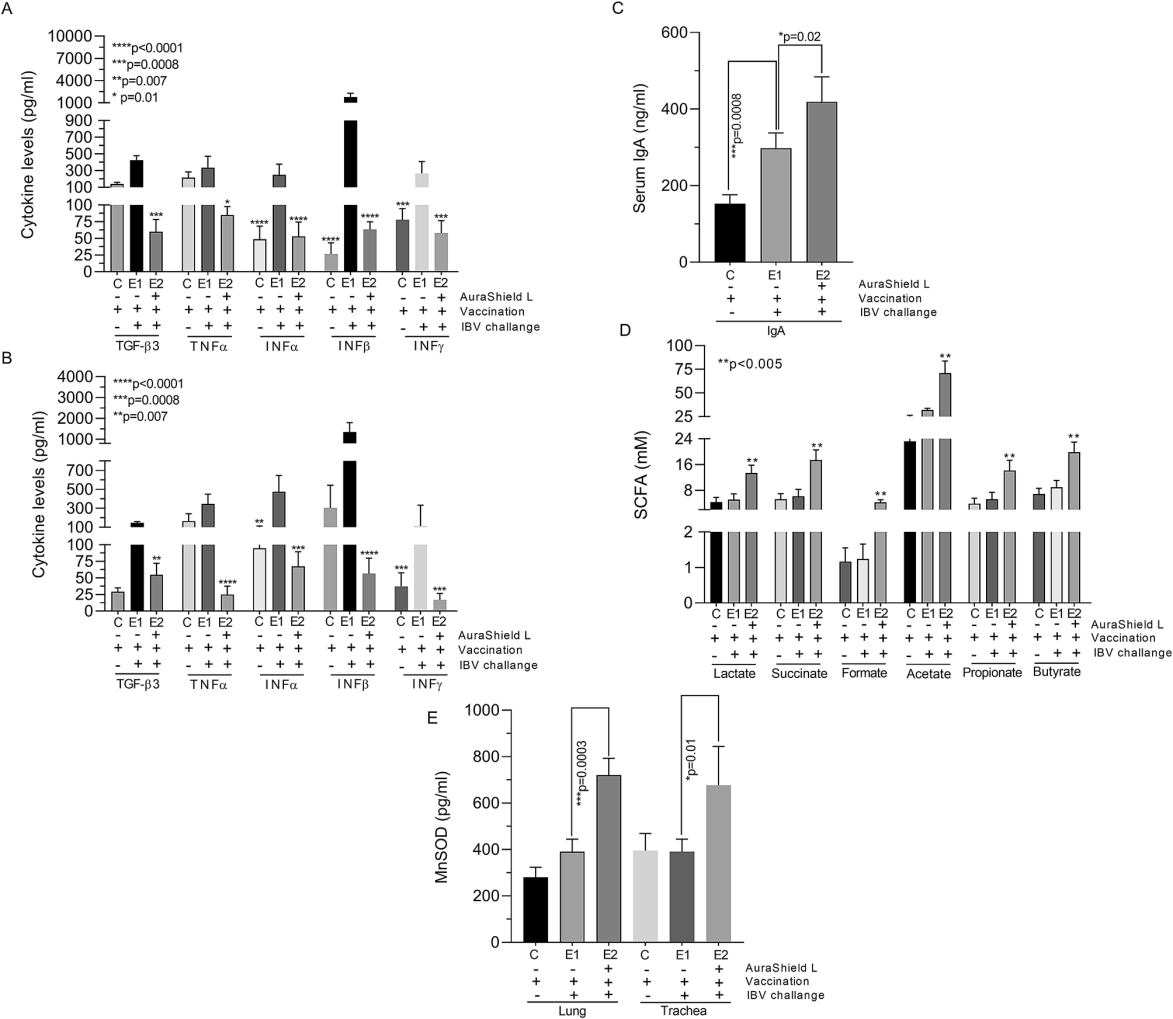
Pro-inflammatory cytokine (TGF-β3, TNF-α, INFα, INFβ and INFγ) production in the trachea and lung tissue of B1648 infected/un-infected broilers which received or not AuraShield L in the drinking water for 5 days post-infection, (**A**) in the trachea and (**B**) in the lungs. (**C**) Represents the IgA levels in broilers serum, (**D**) the SCFAs concentrations in the cecal contents. The levels of MnSOD (pg/ml) in the lung and trachea following exposure to AuraShield L are presented in (**E**). *Asterisks* indicate significant differences with the respective *P* values being indicated on the graph. *Error bars* represent the standard deviation of means from three different experiments. Student *t* test was used to calculate significance (**p < 0.005).

In order to gain more information on why we detect low levels of infection in the trachea and lungs of the experimental group we have designed experiments to investigate the effect against the main pro-inflammatory cytokines, on the immune system and cellular oxidation. The inclusion of AuraShield L in the drinking of the infected broilers (E2) reduced the levels of pro-inflammatory cytokines TGF-β3 (*p* = 0.0008), INFα (*p* = 0.01), INFβ (*p* < 0.0001) and INFγ (*p* = 0.0008) in the trachea (Fig. 2A). Similarly, to trachea, all pro-inflammatory factors in the lung were also decreased by the inclusion of AuraShield L compared to the control and group E1 (Fig. 2B). The significance was calculated against group E1 in order to account for the effect of AuraShield L. Overall inclusion of AuraShield L in the drinking water of group E2 broilers led to a reduction in the expression levels of all pro-inflammatory cytokines investigated indicating that this antimicrobial mixture could have impacted the lung and tracheal inflammation caused by infection. High serum IgA concentrations were detected at 5 days post AuraShield L inclusion in the drinking water (Fig. 2C). Secreted IgA levels were higher (405 vs 300 ng/ml, *p* = 0.02) in group E2 compared to group E1 and moreover, in group E1 (*p* = 0.0008) were significantly higher compared to group C. This is probably a result of vaccination and the response of infection, but equally AuraShield L treatment in group E2 boosted IgA levels above E1 (*p* = 0.02).

We have next investigated the association between SCFA production and manganese superoxide dismutase (MnSOD) expression as indicators of an improved immune response during viral infections. The levels of short chain fatty acids (SCFA) produced in broilers gut (cecum) were also investigated due to their association to a better immunity. The total measured SCFA levels in the cecal contents were significantly increased in group E2 (*p* < 0.01) with the pH levels remained similar between the three groups (Table 2). Succinic acid, lactic acid and butyric acid concentrations were significantly increased by inclusion of AuraShield L (*p* < 0.05) (Fig. 2D). The concentrations of lactic acid, succinic acid, formic acid, lactic acid and butyric acid were significantly increased following inclusion of AuraShield L in the drinking water (*p* < 0.05). Inclusion of AuraShield L in the drinking water of the infected broilers resulted in a significant increase in pulmonary and tracheal MnSOD activity (Fig. 2E). The levels of MnSOD, in the lung increased significantly from 380 pg/ml to 720 pg/ml (*p* = 0.0003) and to 676 pg/ml (*p* = 0.01) in the trachea following the inclusion of AuraShield L. These findings indicate that increased MnSOD expression in the pulmonary and tracheal tissue contributes to a better immune response and more efficient virus elimination.

**Table 2:** Effect of AuraShield L on total SCFA production and pH levels in the cecum (mg/cecum).

Analysis	Groups			*p* value
	C	E1	E2	
AuraShield L	−	−	+	
Vaccination	+	+	+	
IBV challenge	−	+	+	
Total SCFA	0.27 ± 0.19	0.43 ± 0.33	1.45 ± 0.27	< 0.01
pH	7.3 ± 0.01	7.01 ± 0.03	7.4 ± 0.04	ns

*SCFA* short chain fatty acids.

### AuraShield L reduces the clinical signs of disease in broilers challenged with the avian bronchitis virus (B1648) and reduces mortality

In order to quantify the beneficial effects described above we have closely monitored the clinical signs in the challenged broilers during AuraShield administration. Symptoms were mild in both groups except for tracheal rales which impacted 33% of the E1 broilers and 24% in E2 (Table 3). Pulmonary rales were the second obvious clinical sign measurable in the first day of investigation with 11% of broilers affected in group E1 and 18% in group E2. Beginning on day 2 clear signs of improvement were observed in AuraShield treated group (E2). The efficacy of AuraShield L treatment was compared between group E1 and E2 on day 5 and excluded the negative control C group. Broilers displaying no signs of ruffled feathers increased by 150% in group E2 when AuraShield L was included in the drinking water. Sneeze and coughs behaved similarly and demonstrated that the number of broilers showing no clinical signs increased 129.4% and 136.8%, respectively in group E2. Similarly, AuraShield L treatment decreased the number of broilers with tracheal and pulmonary rales. Dyspnoea was lower in the AuraShield L treated group (7% vs. 17%, respectively). No clinical nephritis were recorded during the course of experiment in any group, and clinical symptoms were detected in a small percentage of control broilers, but these were levels higher than that of group E2. The absence or presence of these clinical signs can also be reflected in the mortality rate recorded during the experiment. The inclusion of AuraShield L in the drinking water of broilers in group E2 maintained the broiler survival rate to 93.7%. In contrast, broilers in group E1 had a survival rate of 66.6% and the control group 87.5% (Fig. 1c). The addition of AuraShield L in the drinking water also resulted in performance recovery in the infected broilers (supplementary Table 2).

**Table 3:** Clinical signs in infected and uninfected broilers treated with AuraShield L.

Group	Day	Ruffled feathers	Sneeze	Cough	Tracheal rale	Pulmonary rale	Dyspnoea
		% of broilers with symptoms					
C	1	0	4	3	4	2	3
	2	0	3	2	4	3	4
	3	4	7	8	6	12	4
	4	9	6	8	7	15	7
	5	14	7	10	12	22	5
E1	1	0	6	7	33	11	6
	2	2	8	8	45	23	22
	3	6	12	14	44	12	27
	4	12	11	14	22	25	15
	5	28	14	16	31	28	17
E2	1	0	0	0	24	18	0
	2	1	1	1	9	12	1
	3	6	2	4	2	4	4
	4	4	2	3	2	5	4
	5	4	3	3	2	4	7

## Discussion

Ensuring animal health and welfare also includes having efficient technologies to reduce the incidence of infectious diseases in order of safeguarding of national and international food supplies^19^. A wide range of natural antimicrobial substances have been investigated and have shown activity against various viruses^20^. Organic acids, including citric acid, are known not only for their role in increasing feed utilisation efficiency but also for their role in improving the immune response in infected animals^21^. In this study, we have tested a mixture of natural antimicrobials containing citrus extract, maltodextrin, sodium chloride, lactic acid, and citric acid (AuraShield L) to test its activity in vitro against viruses responsible for avian infectious bronchitis, Newcastle disease, avian Influenza, porcine reproductive and respiratory syndrome (PRRS), and bovine coronavirus. In vivo the antimicrobial mixture was also tested for its ability to reduce the disease signs of infectious bronchitis using a broiler infection model.

Viruses need to penetrate into the cellular cytosol by breaking the endosomal membrane requiring undamaged surface structures to cause infection^22^. AuraShield L acts upon the host and on the pathogen directly indicating a possible disruption of the early stages of viral infection. Experiments developed to evaluate the toxicity of AuraShield L indicated a moderate toxic behaviour towards viruses in cell cultures with the effective cytotoxicity being reached between 0.004–0.007 (µl/ml). Our study shows that the mode of anti-viral action was greatest when cells were treated with AuraShield L before infection or when viruses were incubated with non-cytotoxic concentration of AuraShield L prior to infection. We have also observed that AuraShield L maintained its antiviral properties during adsorption or after penetration into the host cells.

Chickens are an important natural host of the infectious bronchitis virus which targets the upper respiratory tract, and intensive virus replication leads to signs characteristic of the clinical manifestation of this respiratory disease^23^. When AuraShield L was included in the drinking water of artificially IBV (B1648) challenged chicken broilers, the viral mRNA was detected in the trachea and lungs of IBV infected broilers but was significantly decreased by AuraShield L treatment. The reduced presence of IBV virus in trachea of infected birds confirmed the ability of AuraShield L to reduce respiratory disease progression^24^.

AuraShield L treatment reduced the expression of pro-inflammatory cytokines during infection. The transforming growth factors (TGF-β) are polypeptides with role in cell growth and differentiation, and all five examined had similar biological activities including important pro-inflammatory and immunoregulatory activities^25^. In our study the impact on TGF-β3 expression in AuraShield L treated broilers was also significant in both tracheal and lung tissue, with a similar pattern observed for most of the measured cytokines. The detrimental effect on pro-inflammatory cytokines can also be explained by the presence of maltodextrin in AuraShield L, as it has been shown that maltodextrin increased IgA production, an antibody that plays a crucial role in mucous membranes immune function^26^. In our experiments, IgA levels were significantly increased in the serum of broilers treated with AuraShield L and offer protection to the host^27^. The anti-infectious role of IgA was observed in the case of broiler coccidiosis where it has an essential role in the immune response to *E. tenella*^28^. Overall, the levels of pro-inflammatory cytokines were reduced by AuraShiled L to the level of the un-infected control emphasizing its anti-inflammatory effect.

Intestinal immunity if often involved in shaping lung immunity and preventing lung inflammation^29^ through production of short chain fatty acids (SCFA). SCFA’s are known for their role in reducing allergic airway inflammation^30,31^. This anti-inflammatory effect of SCFAs against viral infections was specifically described for the equine herpesvirus 1 (EHV1) where the effect was observed at physiological levels^32^. In our study the inclusion of AuraShield L in the drinking water significantly increased the SCFA (lactate, succinate, formate, acetate, propionate, and butyrate) production which justifies the observed effect on the immune system with positive effects on the viral pathogenicity. Another reason for the observed stimulated immune response in our study was the increased level of manganese superoxide dismutase (MnSOD) in the lung and tracheal tissue. It has been shown previously that in infectious bronchitis (IB) the oxidative status is improved by increasing the levels of manganese superoxide dismutase (MnSOD) with a significant impact on virus elimination via an increased immune response^33^. A similar effect was observed in infections caused by influenza A virus where inclusion of MnSOD in aerosols led to a mild improvement of the disease. Other natural antimicrobials are also known for their antioxidant effect^12^ also when included in the feed of farmed poultr^34^ with a direct effect in controlling the levels of MnSOD^35^.

At present, the primary method of protecting chickens from infectious bronchitis is through vaccination^36^. However, vaccination does not eliminate all clinical signs, which can have detrimental impact on production efficiency, economic return, and animal welfare^37^. Vaccination of broilers coupled with inclusion in the drinking water of AuraShield L reduced clinical signs associated with infectious bronchitis and reduced mortality rates.

In countries with intensive poultry production the incidence of infectious bronchitis virus (IBV) is very high with vaccination only partially diminishing the occurrence of infection and appearance of clinical signs^38^. Identification and testing of novel and natural antimicrobial mixtures designed to reduce the severity of clinical manifestations and possibly to prevent viral infections are crucial for the industry to enhance animal health and welfare, while maintaining performance. Our study clearly shows that antimicrobial compounds and mixtures, such as AuraShield L, can reduce viral pathogenicity in vitro and can boost the efficacy of vaccination in vivo by boosting the immune system through a mechanism that involves SCFA production and increased MnSOD expression.

## Material and methods

### Viruses and antimicrobial mixture

Infectious Bronchitis (B1648)^39^, Newcastle disease (ATCC, 699), Avian Influenza (H9N2, ATCC, VR-1642)^40^, porcine reproductive and respiratory syndrome (ATCC, VR-2386) and bovine coronavirus (ATCC, VR-874) were used. Viruses were propagated in pathogen free 10 days old embryonated chicken eggs. The viruses from the second passage were used in the present experiments. After 48 h post-infection the allantoic fluids were harvested by low-speed centrifugation and stored at − 70 °C. In this study we have used a commercial product (AuraShield L), a mixture of maltodextrin (4%) and sodium chloride (0.5%). As additives lactic acid (30%) and citric acid (15%) and citrus extract (8%) were also included and the balance to 100% is made up with dH_2_O. This commercially available mixture is identified in the manuscript AuraShield L supplied by Auranta LTD.

### Cytotoxicity assay (CC_50_, EC_50_ and SI)

AuraShield L was used for the determination of EC_50_ and SI (selective index). The mixture was titrated from 1 to 1:128 CC_50_ and used for virus exposure. After the addition of 10 μl MTT reagent (Sigma Aldrich) the samples were incubated for 4 h at 37 °C. The EC_50_ concentrations were calculated against extract concentrations.In order to determine the cytotoxic activity of AuraShield L the antimicrobial product was dissolved in water and added to the cell culture medium at a final concentration of 2% in order to quantify the effect on monolayer culture. The Hep-2 cells (ATCC CCL-23) were grown in Dubleco Minimum Essential Medium (DMEM—Sigma-Aldrich) supplemented with foetal bovine serum to a final concentration of 10%. Cells were incubated 37 °C with the medium being renewed every 48 h. The Hep-2 cells were grown in 0.001–0.2% AuraShield L containing medium. The antimicrobial mixture was tested in triplicate and on three different occasions. The absorbance of each well was measured at 620 nm in a microplate reader (Fluostar Omega, BMG Labtech) and the percentage of cell survival was calculated.

### Direct plaque assay

The plaque reduction assay was performed as previously described^41^. Brielfy, 2 × 10^3^ plaque forming units were incubated with different concentrations of AuraShield L for 2 h at room temperature. Serial dilutions of the treated viruses were adsorbed to the cells for 2 h at 37 °C. All assays was performed in triplicates. Incubation was performed for 4 days at 37 °C, followed by 10% formalin fixation. Plaque counting was performed after staining with 1% crystal violet.

### In vitro antiviral activity

Antiviral activity was performed as previously described^41^ except that in our experiments Hep-2 cell line was used. Briefly, Hep-2 cells were pre-treated with AuraShield L before viral infection and separately the viruses were incubated with AuraShield L before infection. In order to test for viral absorption or penetration the cells and viruses were incubated together. AuraShield L was always used at the nontoxic concentration of 0.09% and was added to the cells followed by incubation for 2 h at 37 °C. The supernatant was removed, cells were washed and infected with each virus individually. In the case when the antimicrobial mixture was used to pre-treat the viruses the incubation took place for 1 h at room temperature followed by infection of cells. The absorption period was analysed by mixing individually the viruses with 0.09% AuraShield followed by infection of cells. In the case of replication after infection the inoculum was removed and replaced with media containing 0.5% methylcellulose. Each assay was performed in three replicates on 3 different occasions. Plaque reduction assays were carried out as mentioned above and number of plaques of AuraShield L treated cells and viruses were compared to untreated controls. Data were subjected to statistical analyses using the Graph Prism software package.

### In vivo infections with IBV B1648

**Figure 3:**
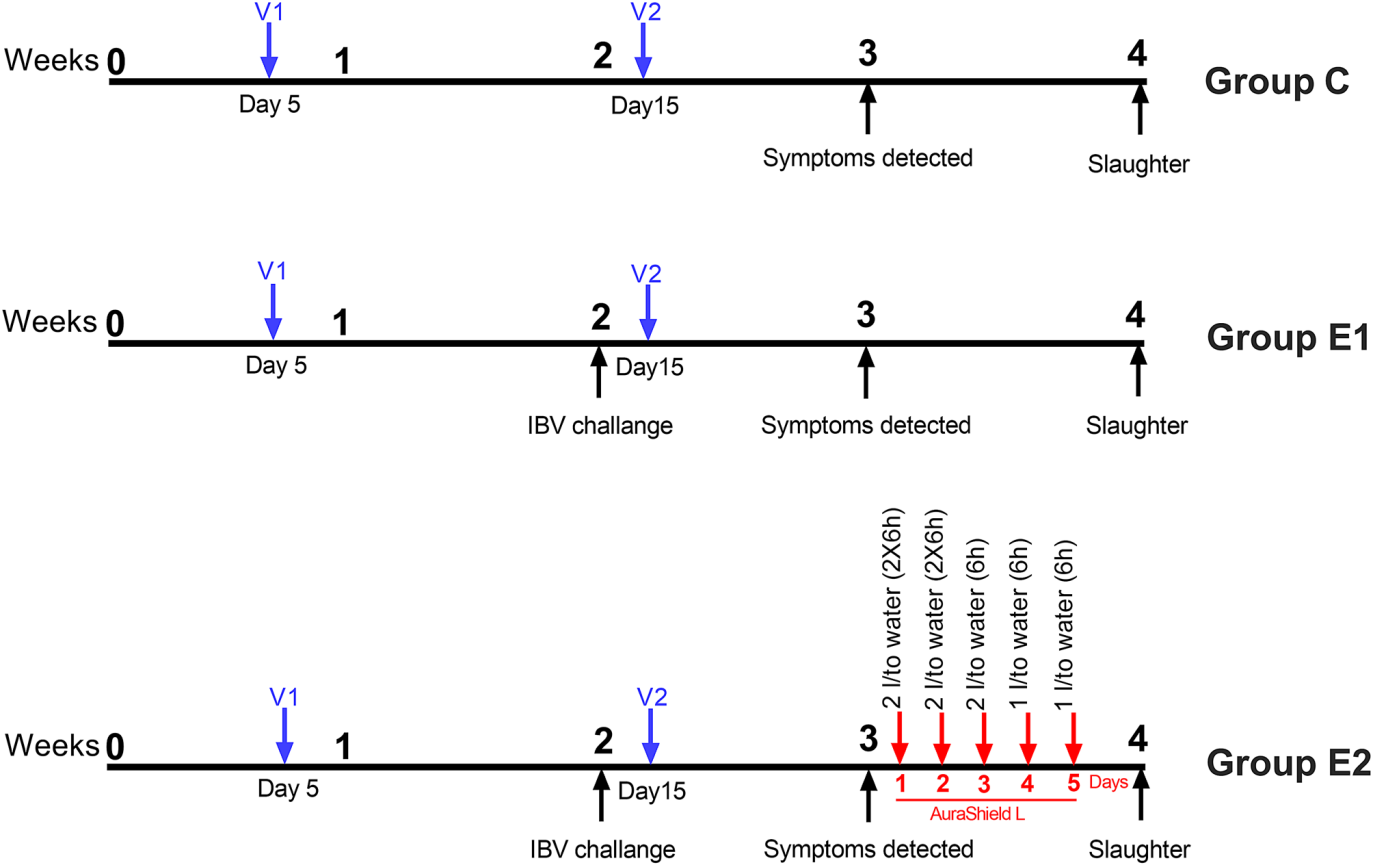
Schematic outline of the in vivo experimental design. Broilers were vaccinated at day 5 (V1) and day 15 (V2). Groups E1 and E2 were challenged with IBV B1648 day 14. Group E2 received AuraShield L in the drinking water for 5 days. At day 28 performance was recorded and 30 broilers euthanized for analysis.

Two-week-old ROSS 308 broilers were separated in three groups (n = 150 broilers/group): Control (C), first experimental group (E1) included broilers challenged with IBV B1648 and the second experimental (E2) included broilers challenged with IBV B1648 and supplemented with AuraShield L as described in Fig. 3. All groups were vaccinated against IBV with Biovac H120 (V1) and Nobilis IB491 (V2). Each broiler in groups E1 and E2 was inoculated intratracheally with (200 μl) with 10^3^ EID50/400 μl B1648 on day 14. AuraShield L was administered to the experimental group E2 through drinking water following the protocol described in Fig. 3. Birds were housed in separate negative pressure isolation rooms, and drinking water and feed were ad libitum duration the experiment. The aim of our study was to assess the effect of Aura Shield L on infection rate and symptoms, and this use of a low IBV challenge dose keeps a low mortality rate as previously described^39^. Control broilers were inoculated with 200 µl PBS (Phosphate Buffer Saline) and served as a negative control group. At 28 days, 30 chickens from each group were humanely euthanized and tracheal and lung samples were stored at − 70 °C for viral RNA quantification by RT-qPCR. Pro-inflammatory cytokines (TGF-β3, TNF-α, INFα, INFβ, INFγ) in tissue of trachea, lung of IBV B1648-infected chickens was determined by using a commercially available enzyme-linked immunosorbent assay systems from Quantikine, R&D Systems. Results of cytokine concentration were expressed as pg (picograms) per milliliter of culture medium. Each study run was performed in triplicate. The experimental design was evaluated and approved by the Ethical and Animal Welfare Committee of the Banat University of Agricultural Sciences and Veterinary Medicine, King Michael I of Romania, Timisoara and we confirm that all methods were performed in accordance with the relevant guidelines and regulations.

### Scoring and clinical signs

Clinical signs, such as ruffled feathers, sneezing, coughing, tracheal and pulmonary rales and dyspnoea were recorded daily during the 5 days of AuraShield L administration and once infection was established. Clinical signs and gross lesions were recorded. Clinical signs were quantified and expressed as a percentage of the total number of birds. The percentage difference (Eq. 1) was used to indicate the efficiency of AuraShield L in reducing the clinical signs of disease.

1 $$\%\, difference = \frac{V1 - V2}{{\left[ {\frac{V1 + V2}{2}} \right]}} \times 100$$

### RT‑qPCR assay for ORF 1a gene

As previously described^39^ RT-qPCR primers were used to detect the B1648 strain in the tracheal samples using the following primers cDNA_FW ggtgttaggcttatagttcctcag, cDNA_RV taaacattagggttgacaccagt, T7_FW taatacgactcactatagggggtgttaggcttatagttcctcag, qPCR_FW gctattgtagaggtagtgtatgtgag and qPCR_RV agggttgacaccagtaaagaat. Viral RNA was extracted from tracheal secretions using the RNeasy Plus Mini Kit (Qiagen, United Kingdom). The RNA was reverse transcribed using Transcriptor First Strand cDNA Synthesis Kit (Roche) according to the manufacturer’s protocol. The mRNA levels were determined by quantitative RT-PCR using QuantiNovaSYBR Green PCR Kit (Qiagen, United Kingdom) on a LightCycler 96 (Roche). The conditions for genes consisted of incubating at 95 °C for 8 min were followed by 40 cycles, each 10 s at 95 °C and 60 s at 58 °C. A total of 5 μl of SYBR Green master mixture was used in each reaction along with 0.5 μl of 10 μM primer mixture, 3 μl of molecular grade water, and 1 μl of DNA sample. Viral RNA extracts from tracheal secretions were analysed in triplicate RT-qPCR reactions as described above. Quantification of the number of viral RNA copies in tissues (RNA copies/g) was similar like in other samples.

### Determination of IgA levels in the serum

The total levels of IgA were measured by IgA Enzyme-Linked Immunosorbent Assay (ELISA) kit (ab157691 ABCAM) according to the manufacturer’s instructions.

### SCFA determinations

The SCFA were analysed by gas chromatography as previously described^42^. In brief, 1 g of ceca was mixed 1 ml of H_2_O and 1 ml of 20 mmol/l pivalic acid solution as an internal standard. The solution was mixed and 1 ml of HClO_4_ (perchloric acid) was added in order to extract SCFA by shaking by vortexing for 5 min. The HClO_4_ acid was precipitated by adding 50 ml of 4 mol KOH into 500 ml of supernatant. The addition of saturated oxalic acid, at 4 °C for 60 min, and centrifugation at 18,000 g for 10 min. Samples were analysed by gas chromatography using SCION-456-GC with a flame ionization detector.

### Superoxide manganese dismutase (SOD2/MnSOD) determination in the lung tissue

The superoxide manganese dismutase was quantified in the lung and trachea using the chicken SOD 2 Eliza Kit (Assay Solution) according to the manufacturer instructions. Briefly, the tissues were rinsed in ice-cold PBS (0.02 mol/l, pH 7.0–7.2) to remove excess blood, minced the tissues to small pieces and homogenized them in a certain amount of PBS and stored at -80 °C until further use. A standard curve was prepared for each experiment. Volumes of 100 μl sample and standard were added per each well in a 96 well plate, except the control well where 0.1 ml of the sample diluent was added. Each well was washed with wash buffer two times for a total of three washes. After the last wash the wash buffer was removed by aspirating or by inverting the plate and blotting it against clean paper towels. In the next step 100 μl of the detection antibody was added to each well followed by incubation at room temperature for 2 h. Next, streptavidin-HRP (100 μl) was added to each well and incubated at room temperature for 30 min. Each measurement was done in triplicate.

### Statistical analysis

Statistical analyses were performed using GraphPad software. Data were represented as mean ± SD. p-values < 0.05 were considered statistically significant (*p < 0.05; **p < 0.01; ***p < 0.001). ANOVA and Student *t* were used. An ANOVA was performed to analyse the percentage decrease compared to control using both Two-way ANOVA (*p* < 0.0001) and Three-way ANOVA (*p* < 0.0001).

## Supplementary information

Supplementary Tables.
