# Supplementary material for: Antiviral activity of a novel mixture of natural antimicrobials, in vitro, and in a chicken infection model in vivo
Source: Sci Rep. 2020 Oct 6;10:16631. doi: 10.1038/s41598-020-73916-1 (PMC7538884; doi:10.1038/s41598-020-73916-1)
Supplement: Supplementary file 1 — Supplementary Tables. [file 41598_2020_73916_MOESM1_ESM.docx]

**Antiviral activity of a novel mixture of natural antimicrobials, *in vitro*, and in a chicken infection model *in vivo***

Igori Balta^1,2^, Lavinia Stef^3^, Ioan Pet^3^, Patrick Ward^4^, Todd Callaway^5^, Steven C. Ricke^6^, Ozan Gundogdu^7*^, Nicolae Corcionivoschi^1,2,3*^

^1^Bacteriology Branch, Veterinary Sciences Division, Agri-Food and Biosciences Institute, Belfast, Northern Ireland, United Kingdom

^2^Faculty of Animal Science and Biotechnologies, University of Agricultural Sciences and Veterinary Medicine, Cluj-Napoca 400372, Romania

^3^Faculty of Bioengineering of Animal Resources, Banat University of Animal Sciences and Veterinary Medicine - King Michael I of Romania, Timisoara, Romania

^4^Auranta, Nova UCD, Belfield, Dublin 4

^5^Department of Animal and Dairy Science, University of Georgia, Athens, GA, United States

^6^Center for Food Safety, Department of Food Science, University of Arkansas, Fayetteville, AR, United States

^7^Faculty of Infectious & Tropical Diseases, London School of Hygiene and Tropical Medicine, 13 Keppel Street, London, WC1E 7HT, UK

**Email addresses:**

Igor Balta: [igori.balta@gmail.com](mailto:igori.balta@gmail.com)

Patrick Ward: patrickward78@gmail.com

Ioan Pet: [ioanpet@eurofins.com](mailto:ioanpet@eurofins.com)

Lavinia Stef: [lavi_stef@animalsci-tm.ro](mailto:lavi_stef@animalsci-tm.ro)

Todd Callaway:todd.callaway@uga.edu

Steven Ricke: s.ricke@uark.edu

Ozan Gundogdu: ozan.gundogdu@lshtm.ac.uk

Nicolae Corcionivoschi [nicolae.corcionivoschi@afbini.gov.uk](mailto:nicolae.corcionivoschi@afbini.gov.uk)

***** Correspondence: [nicolae.corcionivoschi@afbini.gov.uk](mailto:nicolae.corcionivoschi@afbini.gov.uk) and ozan.gundogdu@lshtm.ac.uk

Agri-Food and Biosciences Institute

18a Newforge Lane

Belfast, BT9 5PX

Northern Ireland, UK

00442890255662

[nicolae.corcionivoschi@afbini.gov.uk](mailto:nicolae.corcionivoschi@afbini.gov.uk)

**Supplementary Table 1**

Chemical composition of basal diet

| Item | Starter | Grower | Finisher |
| --- | --- | --- | --- |
|  | 0–10 days | 11–24 days | 25–28 days |
| Wheat | 54.623 | 57.553 | 61.300 |
| Full fat soya | 12.000 | 12.000 | 12.000 |
| Brazilian GM hipro | 25.000 | 21.000 | 17.000 |
| Lime bulk | 0.717 | 0.700 | 0.500 |
| DCP bulk (18.1% p) | 1.654 | 2.000 | 2.150 |
| Salt bulk | 0.200 | 0.200 | 0.200 |
| Sod.bi-carbonate | 0.199 | 0.166 | 0.162 |
| DL methionine | 0.487 | 0.435 | 0.378 |
| L-lysine | 0.373 | 0.318 | 0.281 |
| Threonine | 0.247 | 0.128 | 0.029 |
| Vitamin+mineral premix | 0.500 | 0.500 | 0.500 |
| Soyabean oil | 4.000 | 5.000 | 5.500 |
| **Calculated composition (%)** | | | |
| ME Kcal/kg | 2999 | 3081 | 3133.8 |
| CP | 23.12 | 21.53 | 20.04 |
| Lys | 1.45 | 1.308 | 1.17 |
| Met+Cys | 1.089 | 0.996 | 0.91 |
| Ca | 0.97 | 0.906 | 0.85 |
| AvP | 0.49 | 0.41 | 0.409 |

**Supplementary Table 2**

Effect of AuraShield L on broiler body weight (g), FI (g), and FCR (feed/gain)

| Items  AuraShield L  Vaccination  IBV challenge | Experiment | | |  | |
| --- | --- | --- | --- | --- | --- |
|  | C  -  +  - | E1  -  +  + | E2  +  +  + | SEM | ANOVA |
| *BW*  Day 28 |  | | | | |
|  | 1490.11 | 1242.2 | 1534.06 | 32.09 | *p*<0.0001 |
| *FI*  Day 28 |  | | | | |
|  | 2046.49 | 1750.16 | 2110.13 | 22.31 | *p*<0.0001 |
| *FCR*  Day 28 |  | | | | |
|  | 1.37 | 1.40 | 1.37 | 0.06 | *p*<0.0001 |
